# Bacterial peptidoglycan levels have brain area, time of day, and sleep loss-induced fluctuations

**DOI:** 10.3389/fnins.2025.1608302

**Published:** 2025-07-16

**Authors:** Erika L. English, James M. Krueger

**Affiliations:** ^1^School of Molecular Biosciences, Washington State University, Pullman, WA, United States; ^2^Integrative Physiology and Neuroscience, Washington State University, Pullman, WA, United States

**Keywords:** sleep, peptidoglycan, muramyl peptide, cytokine, peptidoglycan recognition protein, holobiont

## Abstract

Sleep-inducing bacterial cell wall components isolated from brain and urine of sleep deprived animals were identified as peptidoglycan (PG) and muropeptides in the 1980s. Following host detection of PG/muropeptides, downstream signaling mechanisms include release of effector molecules, e.g., cytokines involved in sleep regulation. Understanding of physiological brain PG changes has remained limited, in part due to the historic difficulties of PG quantitation. Herein, we report murine brain PG levels in multiple brain areas within the context of animals’ rest-wake cycles and after sleep loss. Significant time-of-day changes in brain PG levels occurred in all brain areas; lowest levels occurred during the transition from rest to wake periods, at zeitgeber time 12 (ZT12). Highest levels of PG were in brainstem while olfactory bulb, hypothalamic, and cortical PG levels were lower. After 3 h of sleep disruption, PG levels increased in the somatosensory cortex, but decreased in brainstem, and hypothalamus. After 6 h of sleep disruption, PG increased in the brainstem and olfactory bulb compared to control levels. Further, RNA-seq analyses of somatosensory cortical tissue was used to assess sleep loss-dependent changes in genes previously linked to PG. Multiple PG-related genes had altered expression with sleep loss including PG binding and signaling molecules, e.g., Pglyrp1 and Nfil3. In summary, brain PG levels were dependent on time of day, brain area, and sleep history. Further, sleep loss altered brain gene expression for PG-linked genes. Collectively, these data are consistent with the hypothesis that microbe-host symbiotic interactions are involved in murine sleep regulatory mechanisms.

## Introduction

In 1982, somnogenic muramyl peptides (MPs), components of bacterial cell wall peptidoglycan (PG), were posited to be involved in sleep regulation (Krueger et al., 1982a). Subsequently, sleep and microbes were linked in many ways (Besedovsky et al., 2019; Imeri and Opp, 2009), e.g., sleep loss results in bacteremia (Everson and Toth, 2000), and bacterial infections profoundly alter sleep (Toth and Krueger, 1988). Host sleep patterns affect the composition and function of the gut microbiome (Voigt et al., 2016; Benedict et al., 2016; Poroyko et al., 2016; Bowers et al., 2020; Withrow et al., 2021) and gut dysbiosis occurs with sleep loss and sleep pathologies (Wang et al., 2022; Poroyko et al., 2016). Recently, PGs have gained attention as having important roles in brain and additional behaviors, e.g., anxiety-like behavior (Arentsen et al., 2018), brain development (Arentsen et al., 2017), thermoregulation (Gabanyi et al., 2022), and feeding (Gabanyi et al., 2022), yet a mechanistic understanding of microbe-host dynamics and characterization of the molecules involved requires assessment of brain area changes in PG associated with physiological variation. Both sleep and gut microbiome dynamics display circadian fluctuations (Voigt et al., 2016; Heddes et al., 2022; Yan et al., 2021), however, until recently, a role for microbes in physiological sleep regulation seemed unlikely. This is despite extensive characterization of PG and the structural requirements of its smaller MP building blocks as sleep promoting compounds (Krueger et al., 1982b; Krueger et al., 1984). Thus, heretofore whether spontaneous changes in brain PG levels occurred with sleep physiology remained unknown. Furthermore, although the brain expression of a PG binding protein, now called Pglyrp1, increases during rat (Rehman et al., 2001) and mouse (Oles et al., 2020) sleep loss, little is known of other PG-linked mRNA species changes after sleep loss; herein several cortical changes are described.

## Materials and methods

### Mice

All animal procedures, approved by the Washington State University Animal Care and Use Committee, conformed to National Institutes of Health guidelines. Male, C57/BL6J mice (strain #: 000664) obtained from Jackson Laboratories, Bar Harbor, Maine, were acclimated to our facility prior to experimentation. Mice were maintained in plastic, filter-top cages in a 12-h light/dark cycle [zeitgeber time (ZT) 0–12 light, ZT12–24 dark], at an ambient temperature of 23–24°C and relative humidity of 45%, with food and water available *ad libitum*. Collection of tissues included euthanasia by cervical dislocation, immediate removal, and dissection of brains, then tissues flash frozen in liquid nitrogen and stored at −80°C until processing.

### Sleep recording

Because PGs/MPs and proinflammatory cytokines promote sleep (Krueger and Majde, 2017; Pabst et al., 1999) when determining sleep, it was important to avoid inducing inflammation, e.g., caused by use of surgical implants to place EEG electrodes. Thus, all adult male mice, aged 3–5 months, were introduced to Signal Solutions (Lexington, KY), piezoelectric film sensor-fitted rodent sleep monitoring cages and allowed to acclimate for 7 days. Following cage acclimation, spontaneous sleep was determined during undisturbed conditions from 48 h recordings generated from the PiezoSleep integrated software (*n* = 8, all control groups). Separate groups of acclimated mice were subjected to a sleep deprivation (SD) protocol using the gentle handling technique so as not to unnecessarily stress the mice. Animals were sleep deprived for 3 h from ZT0 to 3 (*n* = 8), or for 6 h from ZT0 to 6 (*n* = 9). Sleep recordings were analyzed using SleepStats 2.1 (Signal Solutions, Lexington, KY) software (Mang et al., 2014; Black et al., 2018).

### PG quantitation

We began quantifying brain PG levels using a silkworm larvae plasma assay (FUJIFILM, Wako Chemicals). Before we were able to fully characterize brain PG levels, FUJIFILM discontinued this assay so we switched to using a commercial mouse peptidoglycan assay (My BioSource) for brain and serum PG quantitation. Our initial measures made with the silkworm assay, though limited, were consistent with published brain PG levels using the same assay (Arentsen et al., 2017) and, patterns of brain area differences were consistent with measures made herein using a mouse peptidoglycan ELISA.

**FIGURE 1:**
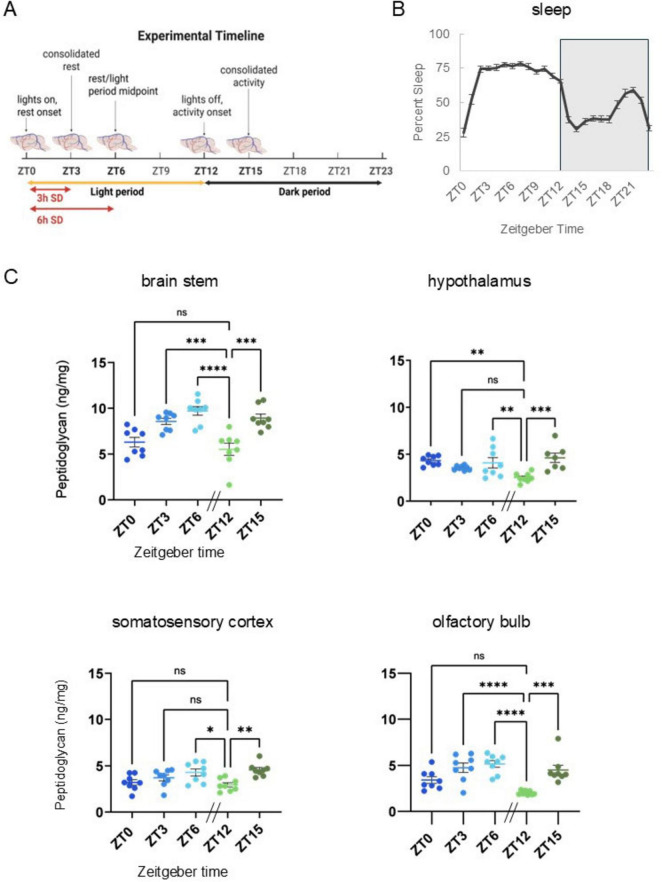
Peptidoglycan levels fluctuate in brain with time-of-day across multiple brain areas and are similar to daily sleep fluctuations. **(A)** Experimental timeline. Brain tissue was collected under undisturbed conditions at times of day corresponding to the animals’ rest-activity patterns and light dark cycles, i.e., zeitgeber time (ZT) 0, ZT3, ZT6, ZT12, and ZT15. [Three and 6 h Sleep amount increased after sleep deprivation (SD) corresponds to Figures 2A–C]. **(B)** Prior to brain tissue collection for PG analyses, spontaneous sleep was recorded for 48 h and hourly percent sleep (2-day average) plotted over ZT for 24 h with ZT labels indicating percent sleep for the hour prior to the ZT timepoint (*n* = 40, i.e., all ZT groups). Patterns of rest and activity correspond to collection times noted above **(A)** and are consistent with patterns typical of C57BL/6J mice. From ZT0 to 12 (light period), mice slept more as compared to ZT12–24 (dark period). Light-dark transitions were associated with dramatic increases (ZT0–3) or dramatic decreases (ZT12–15) in percent sleep, and the increase in percent sleep prior to lights on (ZT18–21) is typical for WT C57BL/6J mice. **(C)** Brain tissue homogenate supernatants were prepared from brain stem, somatosensory cortex, hypothalamus, and olfactory bulb tissues and assayed for peptidoglycan (PG) content (*n* = 8, all brain areas, for all times of day except hypothalamus at ZT15, *n* = 7). PG levels were normalized to brain tissue wet weight (ng/mg). In all brain areas assayed, lowest PG levels were at ZT12, following the murine rest period (ZT0–12). Compared to ZT12 levels, PG levels were increased in all brain areas at ZT15 and ZT6. Data are presented as means ± SEM. [Data from panels **(B,C)** were used to generate correlation coefficients in Supplementary Table 1.] All brain areas except the hypothalamus had similar PG levels and percent sleep changes over time, i.e., increased following lights on, and decreased levels following lights out (ZT12). Error bars indicate mean ± SEM. Ordinary one-way ANOVA with Dunnett’s multiple comparisons test was used to determine group differences for time-of-day PG levels in each brain area assayed. Asterisks denote adjusted *p* values, *≤0.05; **≤0.01; ***≤0.001; ****≤0.0001. Panel **(A)** Created in BioRender. English, E. (2025) https://BioRender.com/3ewyiks.

Timing of brain and sera PG analyses were chosen based on times of day corresponding to light/dark transitions (ZT0/12), following animals’ consolidated rest and activity periods (ZT3 and ZT15), and the rest period midpoint (ZT6) (Figures 1A, B). Following sleep recording, mice were euthanized by cervical dislocation at ZT0, ZT3, ZT6, ZT12, and ZT15 (*n* = 8, all groups). Separate groups of mice were euthanized after SD from ZT0 to 3 (*n* = 8), or ZT0 to 6 (*n* = 9). Cardiac blood, and brain tissues were dissected and immediately processed for serum collection or flash frozen in liquid nitrogen. Frozen tissues were thawed on ice, washed in ice cold, sterile PBS, homogenized via sonication (20 mg tissue per clean, sterile ml of PBS), then centrifuged at 9,500 × *g* at 4°C, and supernatants collected for assay. Whole blood was set at 4°C overnight (as specified in the ELISA kit-supplied protocol) then centrifuged the following morning at 4°C for 15 min at 580 × *g* (2,500 rpm), and serum collected and stored at −80°C until being assayed for PG content. PG levels in brain homogenate supernatants and serum were determined using a mouse, PG ELISA kit (My BioSource, Cat #: MBS263268). As requested, all kits were prepared using the same raw materials, specified by lot number. All assays were performed as described in the kit supplied protocol with endpoint absorbance read using a BioTek Synergy H1 plate reader. PG levels were interpolated from assay standard curves using the kit-supplied PG standard, prepared as a twofold serial dilution series (200 ng/ml to 3.125 ng/ml). Interpolated PG values were corrected for dilution, normalized to tissue wet weight (as ng PG per mg of tissue), or reported as ng/ml for sera.

### RNA sequencing and analysis

We used RNA-seq to investigate mRNA brain changes, following 8 h SD (Figures 3A, B), in the cortex, a brain area in which we observed SD-related change in PG levels. Separate groups of wildtype male, C57/BL6J mice aged 2–3 months, were used to assess somatosensory cortex gene expression profiles of genes previously linked to bacteria detection and signaling. Dissected somatosensory cortical tissues were immediately snap frozen in liquid nitrogen and stored at −80°C until RNA was extracted. Total RNA was extracted using TRIzol reagent and the Qiagen RNeasy Micro Kit (Qiagen, Cat. #: 74004) from somatosensory cortex tissues collected at ZT4 with (*n* = 3) and without (*n* = 3) 8 h prior SD. SD included gentle handling from ZT20 to 4 (Figure 3A). During the 8 h SD, vivarium lighting was maintained on the typical 12 h L/D timing. From ZT20 to 24, SD was performed under “dark” conditions, using dim red light to enable visual verification during SD. RNA preparation, RNA integrity assessment, library preparation and assessment, and paired end sequencing (100 bp) (Illumina HiSeq) was performed as previously described (Lee et al., 2025). On average, for each sample, the yield was 40 million reads. HISAT2 was used (Kim et al., 2015) with the mouse reference genome (mm10, UCSC) to align the RNA-seq data (FASTQ files). Expression quantification and differential expression were analyzed following the protocols as described in Sellegounder et al. (2019). Methods of analysis were specifically chosen for the sample size with inherent low power, and to suit the discrete nature of the dataset, being the use of a single time point to capture physiological response to sleep loss which is a dynamic process. DESeq2 is suited to analysis of datasets with the noted limitations and provides a statistical approach that is based on pooling of information for multiple genes and uses assumptions of gene variance measured within a single experiment to provide a quantitative approach, highlighting the strength of differential expression as opposed to simply the presence of differentially expressed genes (Love et al., 2014). Differential gene expression profiles were generated (sleep disrupted vs. undisturbed controls) (HTseq and DEseq2) using a threshold of adjusted *p*-value < 0.05. Resultant profiles were mined for genes of interest, i.e., genes previously linked to PG.

All differentially expressed genes identified using DEseq2 with an adjusted *p*-value of ≤0.05, were quantified then sorted for up vs. down regulation, and results used to generate a Venn diagram (Figure 3C). This same gene list was used with Reactome 3.7 (database release 92) open-source software to analyze the gene list (using default settings) to identify associated functions of gene groups. Of the 5,501 genes, 1,137 entities were not found, i.e., excluded from functional assignments. The analysis resulted in the identification of 28 associated cellular processes or functional groups. The top 10 gene cluster associations are listed in Figure 3C (order based on the highest ratios, i.e., the number of genes from our list matching the software’s functional gene group lists).

### Statistical analyses

**FIGURE 2:**
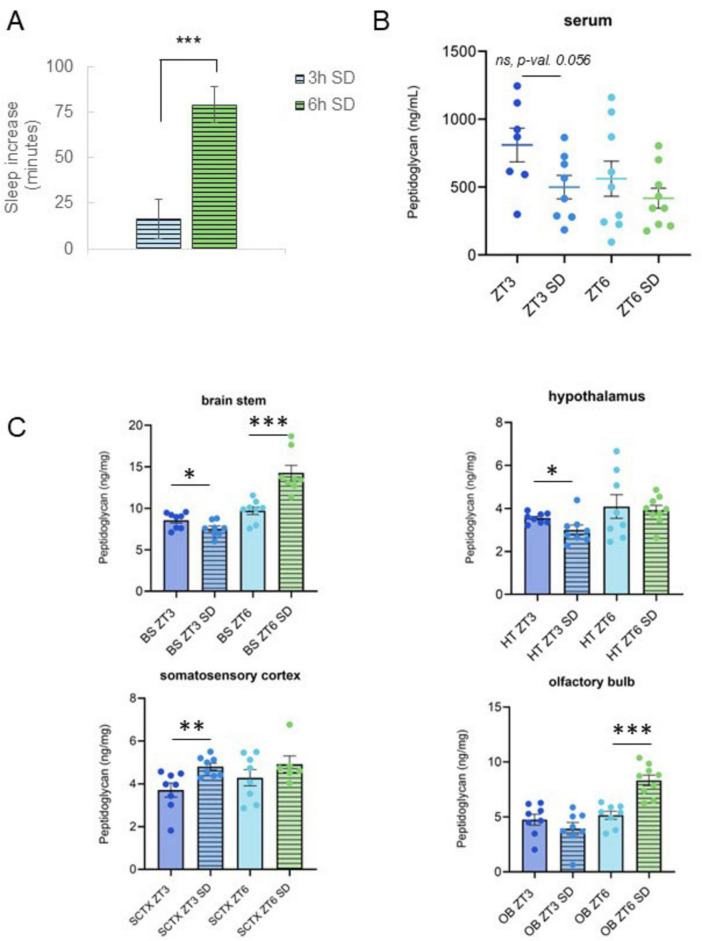
Changes in serum and brain peptidoglycan levels following sleep loss are dependent on sleep loss amount. **(A)** Sleep amount increased after sleep deprivation (SD). Spontaneous sleep (*n* = 8) and sleep following 3 h (*n* = 8) or 6 h SD (*n* = 9) were compared for 12-h periods (from ZT3 to 15, or from ZT6 to 18) and differences (in minutes spent asleep) reported. Mice had a greater increase in sleep following SD from ZT0 to 6 vs. the increase after SD from ZT0 to 3. **(B,C)** Serum and brain PG levels were determined from tissue supernatants prepared from tissues and blood collected at zeitgeber time (ZT) 3 and ZT6 with and without 3 or 6 h SD from ZT0 to 3 or from ZT0 to 6 (Figure 1A). PG levels, normalized to tissue wet weights for brain (ng/mg), are plotted for brain and serum (ng/ml) (note different *y*-axis scales) under control (*n* = 8 all control groups except serum; serum ZT3, *n* = 7, serum ZT6, *n* = 9) and SD conditions [*n* = 8 all SD groups except ZT6 SD; olfactory bulb (OB), hypothalamus (HT), and serum ZT6 SD, *n* = 9, somatosensory cortex (SCTX), *n* = 7]. Following sleep deprivation from ZT0 to 3, PG levels decreased in brain stem (BS) and HT but increased in SCTX. The decrease in serum PG levels following SD from ZT0 to 3 trended toward a significant decrease. Following SD from ZT0 to 6, BS and OB PG increase was significant and the direction of change was reversed as compared to the change following 3 h SD. [Data from panels **(A, B)** were used to generate Supplementary Table 1 correlation coefficients.] Control data shown in Figure 1C. Student’s *t*-tests were used to determine PG level and sleep differences for control vs. SD, and for 3 h vs. 6 h SD groups. Graphs denote group means ± SEM, asterisks indicate *p* values, *≤0.05; **≤0.01; ***≤0.001.

**FIGURE 3:**
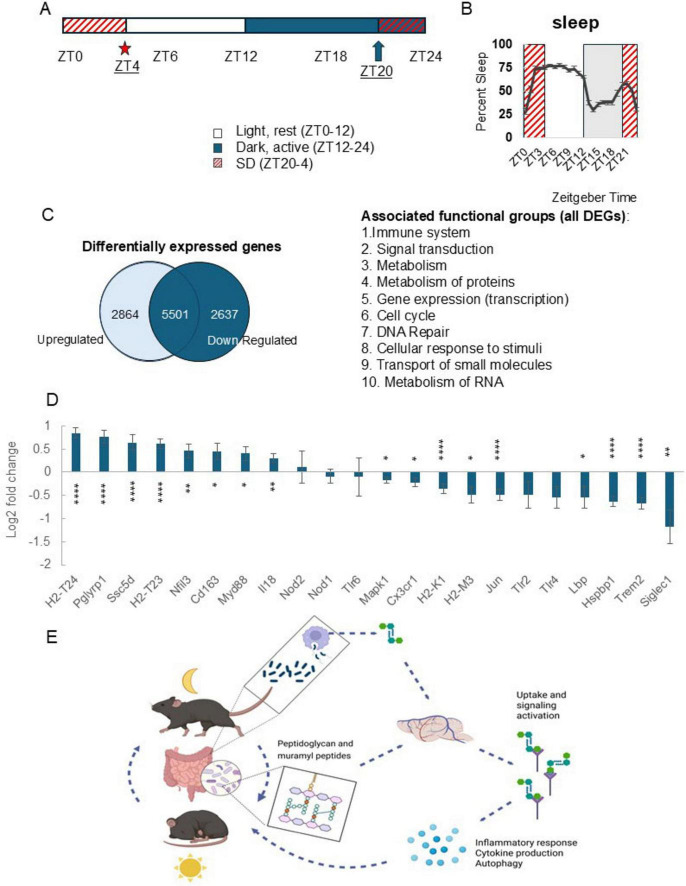
Sleep loss alters cortical expression of genes with known roles in peptidoglycan detection and signaling activity. **(A)** Experimental timeline. Total RNA was extracted from somatosensory cortices collected at zeitgeber time (ZT) 4 under control (*n* = 3) and 8 h sleep disrupted (SD) conditions (*n* = 3) [SD from ZT20 (arrow) to ZT4 (star)]. Total RNA was sequenced using RNA-seq. **(B)** SD from ZT20 to 4 spans the dark to light transition and two periods where sleep amount is highest in the light and dark periods (from ZT0 to 3 and ZT20 to 21); increases typical for C57BL/6J mice. **(C)** Venn diagram showing differential cortical gene expression for control vs. SD groups. Differentially expressed genes were filtered based on significant adjusted *p* values and resultant genes further sorted for up vs. down regulated genes. Of the 5,501 genes which had significant change in expression with SD, 2,864 were upregulated while 2,637 were downregulated. Functional associations of genes were determined using Reactome 3.7 and included immune, signaling, cell cycle, and metabolic activity-linked gene associations. **(D)** Differential gene expression profiles generated in DESeq2, control vs. SD, are plotted as log2 fold change values (adjusted *p*-value, ≤0.05) for genes with known involvement in bacterial cell wall fragment detection and signaling processes. Following SD, expression of peptidoglycan receptor Pglyrp1 was highly upregulated as were genes with functions including antigen presentation and inflammatory signaling, while bacterial cell wall binding proteins Siglec1, Trem2, and Lbp were downregulated. Error bars indicate log2 fold change standard error generated using DESeq2 with the integrated multiple testing adjustments performed with a false discovery rate set at <0.05 and based on the Benjamini–Hochberg procedure. Asterisks denote adjusted *p* values, *≤0.05; **≤0.01; ****≤0.0001. **(E)** Brain peptidoglycan and the rest-activity cycle model. Peptidoglycans are constitutively produced, in part via normal bacterial cell cycle activity and macrophage digestion and released into host circulation (Wheeler et al., 2023; Wheeler and Gomperts Boneca, 2024). Circulating and brain peptidoglycan levels are variable depending on time of day and rest-activity history of the host (Figures 1B, 2A, B and Supplementary Figure 1). Detection of bacterial cell wall components triggers production of inflammatory molecules, including multiple cytokines, e.g., treatment of several different cell types with Pglyrp1 induces production of IL1 and TNF (Bhusal et al., 2024). We propose, following brain detection of peptidoglycans, likely via Pglyrp1 and other peptidoglycan sensing molecules, changes in gene transcription and release of inflammatory molecules includes sleep regulatory cytokines, e.g., IL1 and TNF, which leads to promotion of sleep. Coupled with daily patterns of rest, feeding, and activity, the daily, rhythmic flux of bacterial cell wall fragments into circulation and into the brain, presents a 24 h cycle which is responsive to changes in rest and activity of the host. **(D)** Created in BioRender. English, E. (2025) https://BioRender.com/54uzyx8.

Ordinary one-way ANOVA with Dunnett’s multiple comparisons test was used to determine group differences for time-of-day PG levels (Figure 1C) and unpaired Student’s *t*-tests performed to denote significance in control vs. sleep disrupted peptidoglycan measures (Figures 2B, C). Gene expression analyses were performed in DESeq2 (Lee et al., 2025) and adjusted *p*-values for each gene, as calculated in DESeq2 used to denote significance for control vs. sleep disrupted groups; *p*-values determined with the integrated multiple testing adjustments performed with a false discovery rate set at <0.05 and based on the Benjamini–Hochberg procedure (Figure 3D). Spearman’s correlation calculations (Supplementary Figure 1) were based on raw data values for hourly sleep percentages (Figure 1B) (the hour leading up to each ZT timepoint) generated in SleepStats 2.1 (Signal Solutions, Lexington, KY) and ELISA PG levels (Figures 1C, 2B, C and Supplementary Figure 1) were those interpolated from assay standard curves and normalized to tissue wet weights or serum volume.

### Data availability

RNA-seq raw data, all processed gene quantification files, and differential expression files were deposited in NCBI’s SRA database through the Gene Expression Omnibus (GEO) repository (accessible with accession number GSE263872).

## Results

### Brain PG levels differed between brain areas and changed with daily rest and activity rhythms

Brain PG analyses focused on brain stem, hypothalamus, and somatosensory cortex due to their sleep regulatory functions (Scammell et al., 2017) and included olfactory bulb for its direct contact with microbes (Leyva-Grado et al., 2009). Sampling times were selected based on the 24 h, rest-activity patterns of wildtype mice; ZT0, the transition from the dark to light period and also the transition from active to inactive times of day; ZT3, corresponding to consolidated rest, i.e., immediately following a dramatic increase in sleep (from ZT0 to 3); ZT6, the rest period midpoint; ZT12, the transition from the light to dark period, and the transition from the primary rest to primary active times of day; and ZT15, corresponding to consolidated activity (Figures 1A, B). Spontaneous sleep was highest during the light period (ZT0–12) and lower during the dark period (ZT12–24), with distinct large increases/decreases in sleep amount occurring with light/dark transitions (Figure 1B). Distinct PG levels were found in the various brain areas examined. Within each time-of-day PG levels in brain stem were significantly higher than all other brain areas assayed (determined by one-way ANOVA with Tukey’s multiple comparisons test), i.e., the olfactory bulb, hypothalamus, and somatosensory cortex all had similar lower PG levels (Figure 1C). PG levels varied with time of day and the pattern of change during the animals’ rest period (ZT0–12) mirrored sleep amount (Figure 1B). Thus, in brain stem, hypothalamus, and olfactory bulb PG levels were higher during the light period compared to lowest levels at lights out (ZT12) (Figure 1C). Overall PG fluctuation patterns were similar between brain stem, somatosensory cortex, and olfactory bulb, while hypothalamic fluctuation differed, i.e., levels decreased from ZT0 to ZT12, then rose from ZT12 to ZT15.

### Sleep disruption increased sleep amount in the hours following sleep loss and perturbed brain PG levels

Using separate groups of animals, mice were subject to SD for a mild (3 h, from ZT0 to 3) or moderate time period (6 h, from ZT0 to 6). SD was followed by sleep recording during the recovery period (immediately following, and up to 24 h after SD), or blood and brain tissue collection for PG measurements. Compared to undisturbed sleep, sleep increased post-SD in both mild and moderate SD mice, with a greater increase following moderate SD as compared to mild SD (Figure 2A). Brain PG levels were different after mild vs. moderate SD (Figure 2C) and changed in an area-dependent manner. Brain stem and hypothalamus PG levels decreased during 3 h SD. In contrast, somatosensory cortical PG levels increased while olfactory bulb levels were unchanged after 3 h SD. Conversely, after 6 h of SD, brain stem and olfactory bulb PG levels significantly increased (Figure 2C).

To compare changes in circulating PG with brain PG levels, serum was assayed for PG content (Figures 2B and Supplementary Figure 1). Serum PG levels decreased after 3 h SD while 6 h SD had no effect on PG levels (Figure 2B). Analysis of serum vs. brain area PG amounts at ZT0, ZT3, ZT6, ZT12, and ZT15 indicated brain stem PG levels were correlated (Spearman’s correlation) with circulating (serum) PG levels during the animals’ active hours and at the transition from active to rest periods (ZT12–0) (Supplementary Table 1, I). All other brain areas included in the analysis had low or negligible, insignificant correlation coefficients. Interestingly, the relationship was inverse at the beginning of the active phase (ZT12, immediately following rest), vs. the end of the active phase (ZT0) suggesting physiology, e.g., rest/activity influences brain PG changes.

Given observed changes in brain PG levels during SD (Figure 2C), we assessed whether sleep amount at the rest period midpoint (ZT6) in undisturbed or SD mice correlated with brain PG levels (Supplementary Figure 1 and Supplementary Table 1, II). In undisturbed mice, sleep amount (in the hour prior to ZT6, ZT5–6) was not correlated with PG level in any brain area assayed, or with serum PG. However, during moderate (6 h) SD, somatosensory cortex and hypothalamic PG levels correlated with (disrupted) sleep amount (from ZT5 to 6). Hypothalamic PG levels negatively correlated, and cortex PG levels positively correlated with sleep amount (Spearman’s correlation), further suggesting a brain area specific response to SD (Supplementary Table 1, II and Figure 2C). Collectively, data suggest SD-induced, altered brain PG levels are brain area specific, linking sleep with bacterial cell wall products in brain.

### Changes in cortical brain gene expression after sleep loss includes PG linked genes

Extensive changes in brain gene expression occur during SD reflecting the complex physiological changes which manifest in transitions between wakefulness and sleep (Lee et al., 2025; Figure 3C). As a first step in identifying PG-linked genes altered by SD we used DESeq2 for differential gene expression analyses to employ a rigid set of parameters to generate a quantitative, statistically based profile of differential gene expression values. This method employs pooling variance information across multiple genes within the experiment, employing shrinkage estimation of gene expression dispersion (Love et al., 2014). Gene expression in undisturbed conditions was compared to SD expression profiles to identify sleep affected PG receptors and associated PG-linked signaling genes. SD from ZT20 to 4 (Figures 3A, B) captured the two peaks in sleep percent during a 24 h day typical of C57BL/6J mice, i.e., the beginning of the light period (ZT0–4) and the end of the dark period (ZT20–0) (Figure 3B). SD-induced changes in the cortex included genes with known PG detection and PG signaling associations. Several pattern recognition receptors (PRRs), e.g., Toll-like receptors, nod-like receptors, and Pglyrp1 were included in the analysis. Expression of PRRs Pglyrp1, Ssc5d, and Cd163 upregulated during SD. Among the genes upregulated with SD were immune and inflammatory mediators, e.g., PG receptor Pgylrp1, major histocompatibility complex (MHC) component genes (H2-T24 and H2-T23), scavenger receptors that detect microbial-associated molecular patterns (Cd163 and Ssc5d), and genes linked to microbe-mediated immune function (Nfil3, Myd88, and Il18). Downregulated PG-linked genes included bacterial cell wall component binding molecules (Lbp, MHC components, Siglec1, and Hspbp1) and genes with known roles in signaling events downstream of bacterial product detection (Mapk1, Jun, and Siglec1) (Figure 3D). Collectively, results suggest SD alters many cortical genes with known PG-associations and confirms prior reports that somatosensory cortex Pglyrp1 mRNA increases after SD (Rehman et al., 2001; Oles et al., 2020).

## Discussion

Previously, quantitative brain PG reports were limited to the cerebellum (Arentsen et al., 2017) and to exogenous, radiolabeled PG/MPs injected systemically (Wheeler et al., 2023). More recent literature links bacterial cell wall products, e.g., PGs, to host physiology (Arentsen et al., 2017; Gabanyi et al., 2022; Kala et al., 2023; Wolf, 2023) but lacks description of daily endogenous PG dynamics in brain. Here, sleep/time of day measures were coupled with quantitative measurement of endogenous PG in brain, linking brain area-specific PG changes with animals’ rest and activity cycles. We demonstrate brain PG level is dependent on the time of day, brain location, and sleep amount in healthy adult mice (Figures 1C, 2C). Although the results presented herein are consistent with the hypothesis that brain PG levels play a role in sleep regulation, they are mostly descriptive. However, they are required to understand the brain mechanisms responsible for sleep regulation developed within the literature over the past 40 years. Thus, PG/MPs induce multiple downstream molecular steps involved in physiological sleep regulation as well as sleep responses associated with inflammation, infection, and other pathologies. Multiple reviews and primary data publications address these molecular mechanisms (e.g., Krueger et al., 2008; Krueger et al., 2013; Imeri and Opp, 2009; Krueger et al., 1984; Rehman et al., 2001; Oles et al., 2020; Krueger and Majde, 2017; Pabst et al., 1999; Lee et al., 2025; Wheeler et al., 2023; Chedid et al., 1984; Bhusal et al., 2024; Shoham et al., 1989; Dinarello and Krueger, 1986; Churchill et al., 2008; Yasuda et al., 2005; Opp, 2005; Laman et al., 2020; Tosoni et al., 2019; Wheeler and Gomperts Boneca, 2024). For example, MPs induce the cytokine IL1 (Dinarello and Krueger, 1986), a well characterized sleep regulatory substance with brain area-specific, time-of-day changes in mRNA levels (Taishi et al., 1997; Taishi et al., 1998). Further, the neuron-specific IL1 receptor accessory protein, called AcPb, but not other AcP isoforms, upregulates during sleep loss (Taishi et al., 2012) and AcPb is required for a normal response to sleep loss, i.e., non-rapid eye movement sleep rebound (Davis et al., 2015). Collectively, current results provide much needed evidence supporting the hypothesis that bacterial cell wall PG, originally proposed in 1982 (Krueger et al., 1982b), has a role in sleep regulation.

Peptidoglycan receptors and other molecules with which PG interacts change during sleep loss (Figure 3D). PGs, their receptors expressed by mammalian hosts, and their roles in inflammation are extensively studied (Dziarski and Gupta, 2006; Krueger and Takahashi, 1997; Bastos et al., 2021; Laman et al., 2020). The PG receptor Pglyrp1 is an inflammatory mediator with roles in neuroinflammatory modulation (Bhusal et al., 2024; Sharapova et al., 2020; Read et al., 2015; Yao et al., 2013). Treatment of multiple cell types with Pglyrp1 induces proinflammatory cytokines including TNF (Bhusal et al., 2024). IL1 and TNF can act locally to induce sleep (Churchill et al., 2008; Yasuda et al., 2005), but also have roles in regulating whole animal sleep, and immune responses (Opp, 2005; Imeri and Opp, 2009). Thus, sleep, PGs, and Pglyrp1 are all implicated in induction of TNF and IL1. Herein, we also extend the search to identify PG-linked genes affected by sleep loss by analyzing cortical gene expression following SD. We focused on the cortex due to the high correlation between sleep amount and cortical PG levels after 6 h SD (Supplementary Table 1, II). Among the sleep-affected genes previously linked to PG, Pglyrp1 transcript levels upregulated in mice confirming prior reports (Rehman et al., 2001; Oles et al., 2020). Among the PG receptors included in our gene expression analysis, e.g., Toll-like and Nod-like receptors, the upregulation of Pglyrp1 with sleep loss suggests a distinct sleep-linked role for Pglyrp1 in brain. It is likely that PG receptors altered with SD play a role in associated brain PG level changes.

In addition to PG receptors, genes with known roles in IL1 and inflammatory signaling pathways, e.g., Nfil3 (Yang et al., 2022; Ohne et al., 2016), Cd163 (Elmquist et al., 1997), Myd88 (Gosselin and Rivest, 2008), Il18 (Boraschi et al., 2023), Trem2 (Gawish et al., 2015), and Jun (Raivich, 2008; Dinarello, 2009; Mantovani et al., 2019) were also included. PG/MPs signal, in part, via cytokines (Chedid et al., 1984; Sharapova et al., 2020). Expression of several genes which make up multiple inflammatory signaling pathways changed with SD. These molecules contribute to cytokine signaling pathways, and pathways which have overlap with Pglyrp1 signaling cascades (Royet et al., 2011; Laman et al., 2020; Bhusal et al., 2024). Changes in gene expression align with observations from previous studies, e.g., in the context of SD, Pglyrp1 has been linked to the well-known sleep regulatory cytokines IL1 and TNF (Oles et al., 2020). Pglyrp1 and TNF transcript levels are highly correlated following SD in mice lacking a neuron-specific IL1 accessory protein (Oles et al., 2020). Taken together, it is possible the increase in brain cytokine levels that occur following SD involves increased Pglyrp1 expression and/or changes in brain PG.

We acknowledge the limitations of a limited RNA-seq dataset used for our analysis. A limited amount of data from which to draw conclusions, together with use of a single timepoint to assess sleep loss driven changes in brain gene expression, led us to carefully consider our interpretation of results. Response to SD occurs over a much broader timescale than that which is captured immediately following the sleep loss event. We have, however, taken careful measures to attend to the inherent limitations, by using multiple corrections-based testing of differential gene expression profiling and a method of analysis suited to analyzing differential expression in small datasets (Love et al., 2014). Thus, we report on a focused group of genes previously linked to PG in the literature, yet it is worth noting that this study was likely not robust enough to capture small effects of sleep loss and thus, we could have missed some differentially expressed genes that may be important in PG sensing and signaling. The endogenous PG levels and degree of change in PG levels reported herein are indeed subtle though statistically significant. It would be of interest to follow up with additional studies which account for time-dependent changes in gene expression following sleep loss and to increase the sample size to increase the chances of capturing responses over time and low-level changes which may have profound effects on neurobiology or brain PG changes.

In the murine gut, microbial gene expression of molecules involved in cell wall synthesis exhibits a rhythmic nature corresponding to the light/dark cycle (Thaiss et al., 2014). The gut is a reservoir for PGs, and translocation and subsequent dissemination into other organs requires an intact gut microbiome (Wheeler et al., 2023). PGs are constitutively released into the host circulation (Wheeler and Gomperts Boneca, 2024) and are found in many organs including the brain (Arentsen et al., 2017; Wheeler et al., 2023). Given the rhythmic nature of many microbe-host interactions (Thaiss et al., 2016; Thaiss et al., 2014; Tofani et al., 2025), we anticipated the fluctuations of PG levels in brain. While similar patterns of daily change in PG levels emerged for several brain areas, brain area PG levels differed between each other and had unique brain area changes following SD (Figures 1C, 2C). Our results are consistent with PG brain amounts following oral delivery of radiolabeled peptides (Wheeler et al., 2023). However, those reports did not describe endogenous PG. Further, previous studies concluded that peripheral and brain PG clearances occur at different rates for different organs (Wheeler et al., 2023; Ladesić et al., 1993), indicating unique regulations. In addition to organ-specific regulation, unique brain-area PG regulation surfaced in our studies.

An interesting aspect of brain PG fluctuations vs. fluctuations in circulating PGs is the discordant patterns in brain vs. periphery with respect to time of day (Figures 1C, 2B and Supplementary Figure 1). It is possible that blood brain permeability and clearance play a role in regulating brain PG entry/exit. Brain permeability and clearance exhibit daily fluctuations (Xie et al., 2013; Zhang et al., 2018; Hauglund et al., 2025). Brain permeability is influenced by the gut microbiome (Braniste et al., 2014) and can be perturbed by peptidoglycans (Roord et al., 1994) and sleep loss (Xie et al., 2013), and blood brain permeability has a role in sleep regulation (Axelrod et al., 2023). Gut permeability is also susceptible to perturbation with sleep loss (Everson and Toth, 2000) and translocation of PG/muropeptides likely precedes dissemination to host organs (Wheeler et al., 2023) which could present unique timing for fluxes in circulating vs. brain PG levels. Thus, the altered PG levels observed following SD and the upregulation of brain Pglyrp1 could be a response to mediate entry/clearance of PGs in the brain. Similar physiological activity has been described for brain clearance. Current understanding suggests brain clearance occurs in a circadian-based fashion, however, the time of day associated with greatest clearance may differ depending on the molecules involved (Xie et al., 2013; Zhang et al., 2018).

While the origins of microbial cell wall products found in brain and the route(s) of entry remains incomplete, the gut is likely a reservoir for both circulating bacterial cell wall components and those found in brain. The requirement of a microbiome for PG dissemination to host organs (Wheeler et al., 2023) highlights the interdependence of host physiology with resident microbes and movement of biologically active cell wall components. While sleep loss and sleep pathologies are associated with altered gut microbiomes (Wang et al., 2022), we are just beginning to understand the importance of bacterial products in the microbe-gut-brain axis, including PGs, especially in the context of sleep regulation. Herein we present a model to link the existing literature on mechanisms of sleep-promoting bacterial cell wall components to sensing and signaling in the mammalian host in the context of sleep (Figure 3E). Understanding of microbe-host driven aspects of sleep physiology ultimately will enrich the sleep regulation literature to encompass the mammalian holobiont condition thereby providing a new paradigm for the evolution of rest-activity cycles of simple organisms to sleep of higher order organisms.

## Data Availability

The datasets presented in this study can be found in online repositories. The names of the repository/repositories and accession number(s) can be found below: https://www.ncbi.nlm.nih.gov/, GSE263872.

## Supplementary material

Supplementary Table 1 Brain peptidoglycan (PG) levels are correlated with circulating PG levels (I) and sleep amount (II) in a brain area specific manner. I. Correlation coefficients are shown for serum PG vs. brain PG levels (ng/ml and ng/mg, respectively) determined with ELISAs (also presented in Figures 1B, C, 2A, B). Correlations coefficients were generated using Spearman’s correlation calculations for individual mice at ZT0, 3, 6, 12, and 15 to determine whether brain area PG was correlated with peripheral PG levels across the 24 h day. Correlation coefficients indicate high and very high (red) positive and negative correlations for brainstem PG vs. serum PG at ZT0, ZT12, and ZT15, i.e., serum PG was correlated with brain stem PG during the animals’ active period [zeitgeber time (ZT) 12–0]. II. Serum and brain PG levels for individual mice were compared to percent sleep in the hour prior to ZT6 (ZT5–6) (for the same mice), with and without sleep disruption (SD) (from ZT0 to 6). Correlation coefficients (Spearman’s correlation) indicated cortex PG amounts and hypothalamic PG amounts were highly positively correlated (red) and moderately (negatively) correlated (red), respectively, with sleep amounts in the final hour of SD while correlation coefficients did not indicate any correlation in undisturbed mice.

Supplementary Figure 1 Peptidoglycan levels in sera do not change significantly with time of day. Sera collected from wildtype mice at ZT0 (*n* = 8), ZT3 (*n* = 7), ZT6 (*n* = 9), ZT12 (*n* = 7), and ZT15 (*n* = 6) was assayed for peptidoglycan (PG) content using a commercial mouse PG ELISA. Levels were interpolated using the assay standard curves and reported as ng/ml. Circulating PG amounts did not change significantly for the times of day assayed and variability within each time point was high as compared to brain area PG levels (Figure 1B). Ordinary one-way ANOVA with Dunnett’s multiple comparisons test was used to determine group differences for time-of-day PG levels and graphs denote means ± SEM.
