# Supplementary figures and images for: Bacterial peptidoglycan levels have brain area, time of day, and sleep loss-induced fluctuations
Source: Front Neurosci. 2025 Jul 16;19:1608302. doi: 10.3389/fnins.2025.1608302 (PMC12307287; doi:10.3389/fnins.2025.1608302)

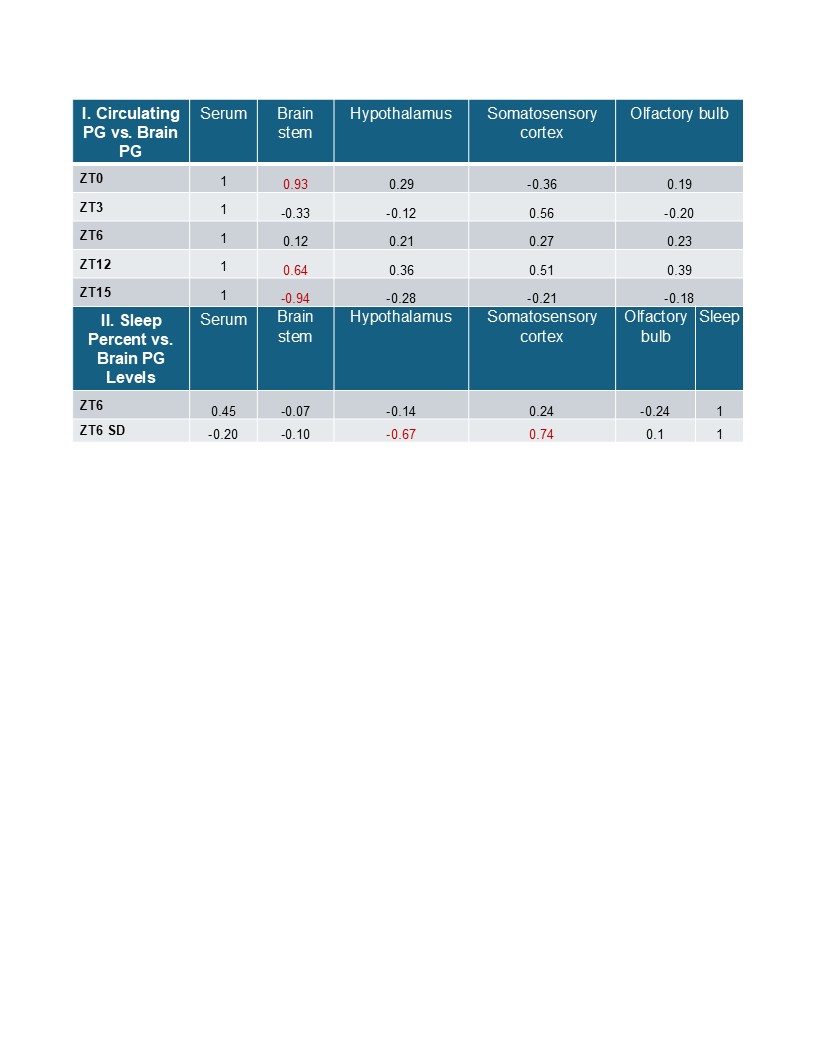

Supplement: Supplementary Table 1 — Brain peptidoglycan (PG) levels are correlated with circulating PG levels (I) and sleep amount (II) in a brain area specific manner. I. Correlation coefficients are shown for serum PG vs. brain PG levels (ng/ml and ng/mg, respectively) determined with ELISAs (also presented in Figures 1B, C, 2A, B). Correlations coefficients were generated using Spearman’s correlation calculations for individual mice at ZT0, 3, 6, 12, and 15 to determine whether brain area PG was correlated with peripheral PG levels across the 24 h day. Correlation coefficients indicate high and very high (red) positive and negative correlations for brainstem PG vs. serum PG at ZT0, ZT12, and ZT15, i.e., serum PG was correlated with brain stem PG during the animals’ active period [zeitgeber time (ZT) 12–0]. II. Serum and brain PG levels for individual mice were compared to percent sleep in the hour prior to ZT6 (ZT5–6) (for the same mice), with and without sleep disruption (SD) (from ZT0 to 6). Correlation coefficients (Spearman’s correlation) indicated cortex PG amounts and hypothalamic PG amounts were highly positively correlated (red) and moderately (negatively) correlated (red), respectively, with sleep amounts in the final hour of SD while correlation coefficients did not indicate any correlation in undisturbed mice. [file Image_1.jpg]

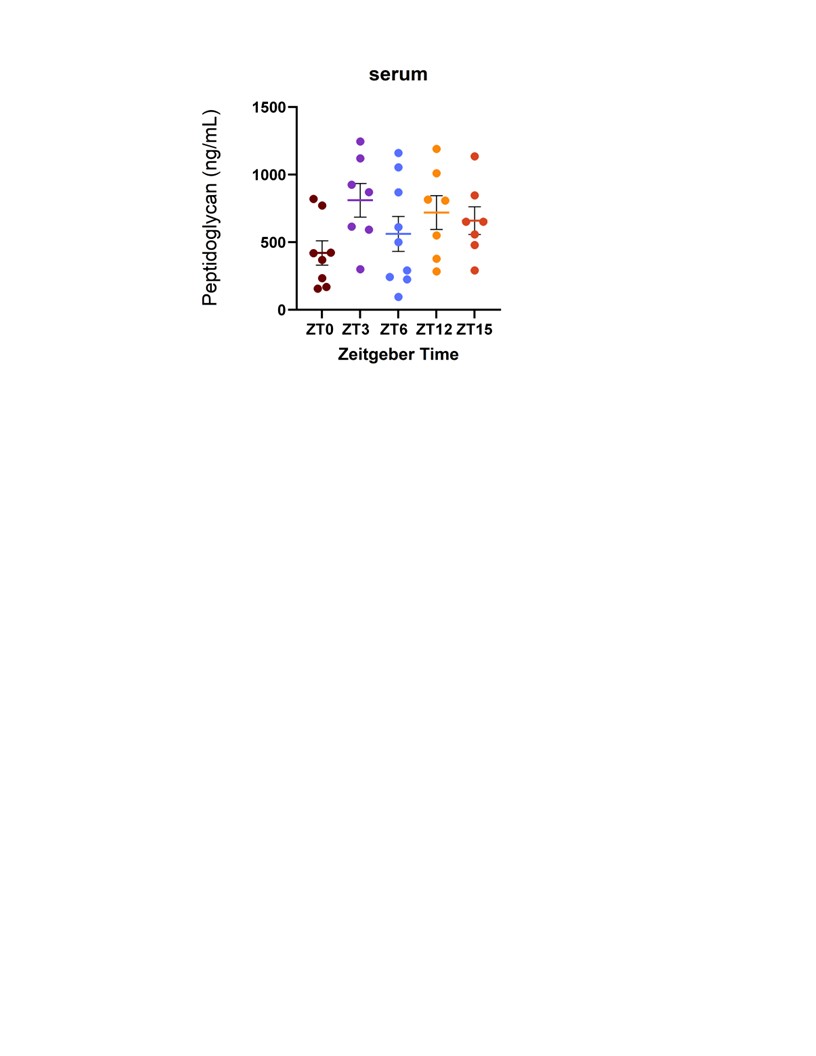

Supplement: Supplementary Figure 1 — Peptidoglycan levels in sera do not change significantly with time of day. Sera collected from wildtype mice at ZT0 (n = 8), ZT3 (n = 7), ZT6 (n = 9), ZT12 (n = 7), and ZT15 (n = 6) was assayed for peptidoglycan (PG) content using a commercial mouse PG ELISA. Levels were interpolated using the assay standard curves and reported as ng/ml. Circulating PG amounts did not change significantly for the times of day assayed and variability within each time point was high as compared to brain area PG levels (Figure 1B). Ordinary one-way ANOVA with Dunnett’s multiple comparisons test was used to determine group differences for time-of-day PG levels and graphs denote means ± SEM. [file Image_2.jpg]
